# 2-(1,2-Dihydro-2-oxopyridin-3-yl)-1,3-benzothia­zol-3-ium bromide monohydrate

**DOI:** 10.1107/S1600536811022847

**Published:** 2011-06-18

**Authors:** Kim Potgieter, Thomas Gerber, Richard Betz

**Affiliations:** a Nelson Mandela Metropolitan University, Summerstrand Campus, Department of Chemistry, University Way, Summerstrand, PO Box 77000, Port Elizabeth 6031, South Africa

## Abstract

The title hydrated molecular salt, C_12_H_9_N_2_OS^+^·Br^−^·H_2_O, the aza-substituted six-membered ring is present as its keto tautomer instead of its aromatic tautomer. The dihedral angle between the fused ring  system and the pyridinone ring in the cation is 6.91 (6)°. In the crystal, bifurcated N—H⋯(O,Br) and O—H⋯Br hydrogen bonds and S⋯O contacts [S⋯O = 3.0526 (10) Å] connect the components into a three-dimensional network. The closest centroid–centroid distance between two π-systems is 3.7420 (7) Å between two benzene rings.

## Related literature

For the crystal structure of 2-(*o*-hy­droxy­phen­yl)benzothia­zole, see: Stenson (1970 ▶); Aydin *et al.* (1999 ▶); Jia & Jin (2009 ▶). For graph-set analysis of hydrogen bonds, see: Etter *et al.* (1990 ▶); Bernstein *et al.* (1995 ▶). For our continuing efforts to create new radio-pharmaceuticals, see: Gerber *et al.* (2011 ▶).
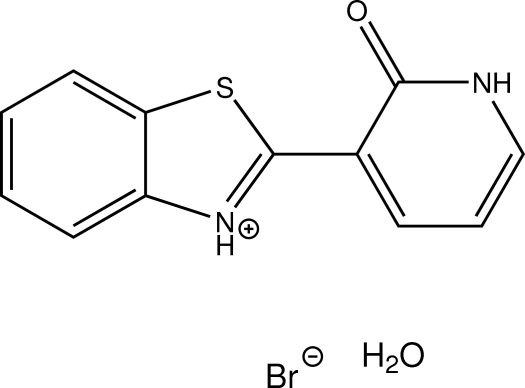


## Experimental

### 

#### Crystal data

C_12_H_9_N_2_OS^+^·Br^−^·H_2_O
*M* *_r_* = 327.20
Triclinic, 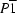

*a* = 5.6480 (2) Å
*b* = 9.9900 (3) Å
*c* = 11.2070 (3) Å
α = 88.808 (1)°
β = 83.098 (1)°
γ = 87.914 (1)°
*V* = 627.25 (3) Å^3^
*Z* = 2
Mo *K*α radiation
μ = 3.44 mm^−1^
*T* = 100 K
0.54 × 0.32 × 0.12 mm

#### Data collection

Bruker APEXII CCD diffractometer
Absorption correction: multi-scan (*SADABS*; Bruker, 2008 ▶) *T*_min_ = 0.825, *T*_max_ = 1.000
11074 measured reflections
3084 independent reflections
3004 reflections with *I* > 2σ(*I*)
*R*_int_ = 0.015

#### Refinement

*R*[*F* ^2^ > 2σ(*F* ^2^)] = 0.016
*wR*(*F* ^2^) = 0.042
*S* = 1.07
3084 reflections
179 parameters
H atoms treated by a mixture of independent and constrained refinement
Δρ_max_ = 0.56 e Å^−3^
Δρ_min_ = −0.28 e Å^−3^

### 

Data collection: *APEX2* (Bruker, 2010 ▶); cell refinement: *SAINT* (Bruker, 2010 ▶); data reduction: *SAINT*; program(s) used to solve structure: *SIR97* (Altomare *et al.*, 1999 ▶); program(s) used to refine structure: *SHELXL97* (Sheldrick, 2008 ▶); molecular graphics: *ORTEP-3* (Farrugia, 1997 ▶) and *Mercury* (Macrae *et al.*, 2008 ▶); software used to prepare material for publication: *SHELXL97* and *PLATON* (Spek, 2009 ▶).

## Supplementary Material

Crystal structure: contains datablock(s) I, global. DOI: 10.1107/S1600536811022847/yk2012sup1.cif

Structure factors: contains datablock(s) I. DOI: 10.1107/S1600536811022847/yk2012Isup3.hkl

Supplementary material file. DOI: 10.1107/S1600536811022847/yk2012Isup4.cdx

Supplementary material file. DOI: 10.1107/S1600536811022847/yk2012Isup4.cml

Additional supplementary materials:  crystallographic information; 3D view; checkCIF report

## Figures and Tables

**Table 1:** Hydrogen-bond geometry (Å, °)

*D*—H⋯*A*	*D*—H	H⋯*A*	*D*⋯*A*	*D*—H⋯*A*
N1—H71⋯Br1	0.89 (2)	2.40 (2)	3.2708 (10)	168.2 (17)
N2—H72⋯O90^i^	0.832 (19)	1.930 (19)	2.7390 (15)	163.6 (17)
O90—H901⋯Br1	0.81 (2)	2.55 (2)	3.3485 (11)	170 (2)
O90—H902⋯Br1^ii^	0.85 (2)	2.49 (2)	3.3360 (10)	176 (2)

Symmetry codes: (i) 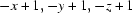; (ii) 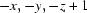.

**Fig. 1.**
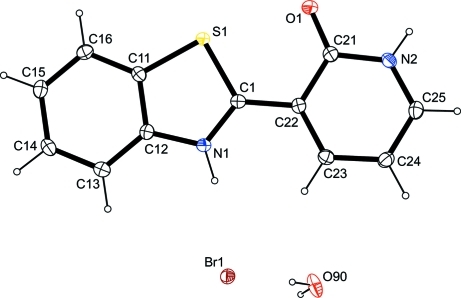
The molecular structure of the title compound, with atom labels and anisotropic displacement ellipsoids (drawn at 50% probability level).

**Fig. 2.**
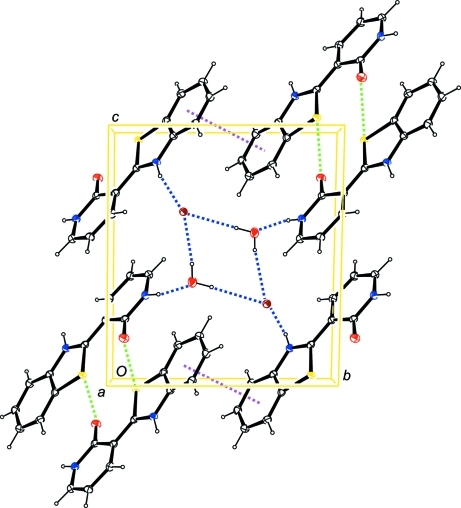
Molecular packing and intermolecular interactions in the crystal structure of the title compound, viewed along [-1 0 0] (anisotropic displacement ellipsoids drawn at 50% probability level). Blue dashed lines indicate hydrogen bonds, green dashed lines S···O contacts and magenta dashed lines π···π interactions.

## supplementary crystallographic information

### Comment

In our continuous efforts to create new radio-pharmaceuticals (Gerber *et al.*, 2011), we attempted the coordination reaction of a bidentate ligand towards a rhenium(V) precursor upon which a crystalline reaction product was obtained. The crystal structure analysis showed the unintentional synthesis of a protonated derivative of the ligand. The structure of 2-(*o*-hydroxyphenyl)benzothiazole is apparent in the literature (Stenson, 1970; Aydin *et al.*, 1999; Jia & Jin, 2009).

In the molecule, the – possible – hydroxy-pyridine moiety is present as its keto-tautomer. Protonation took place on the nitrogen atom of the five-membered heterocyclic subunit. The molecule is essentially flat, the least-squares planes defined by the ring atoms of the benzothiazol moiety and the ring atoms of the hydroxy-pyridine tautomer enclose an angle of only 6.91 (6) °. One molecule of solvent water is present in the crystal structure (Fig. 1).

In the crystal structure, hydrogen bonds as well as S···O contacts (whose range falls by more than 0.2 Å below the sum of van-der-Waals radii of the respective atoms) are present. While the hydrogen bonds originating from the solvent water as well as the protonated nitrogen atom of the five-membered heterocyclic subunit exclusively have the bromide anion as acceptor, the water molecule's oxygen atom serves as acceptor for the hydrogen atom of the intracyclic NH group in the six-membered heterocycle. The pattern formed by the water molecules connecting the bromide anions is reminiscent of a parallelogram (Fig. 2). The S···O contacts give rise to the formation of centrosymmetric dimers. In total, the components of the crystal structure are connected to a three-dimensional network. In terms of graph-set analysis (Etter *et al.*, 1990; Bernstein *et al.*, 1995), the descriptor for the hydrogen bonding system is *DDDD* on the unitary level. The parallelogram shaped pattern necessitates a *R*^4^_2_(8) descriptor on the binary level. The description of the S···O contacts is possible by a *R*^2^_2_(10) descriptor on the unitary level. The closest intercentroid distance between two π-systems was found at 3.7420 (7) Å and was observed between two phenyl-moieties.

The packing of the title compound is shown in Figure 3.

### Experimental

The compound was unintentionally obtained upon reacting ReOBr_3_(PPh_3_)_2_ and the unprotonated title compound in methanol. Crystals suitable for the X-ray diffraction study were obtained upon free evaporation of the solvent at room temperature in the course of three days.

### Refinement

Carbon-bound H atoms were placed in calculated positions (C—H 0.95 Å) and were included in the refinement in the riding model approximation, with *U*(H) set to 1.2*U*_eq_(C). The hydrogen atoms on the water molecule as well as on both nitrogen atoms were located on a difference Fourier map and refined freely.

### Crystal data

**Table:**

C_12_H_9_N_2_OS^+^·Br^−^·H_2_O	*Z* = 2
*M**_r_* = 327.20	*F*(000) = 328
Triclinic, *P*1	*D*_x_ = 1.732 Mg m^−^^3^
Hall symbol: -P 1	Mo *K*α radiation, λ = 0.71069 Å
*a* = 5.6480 (2) Å	Cell parameters from 9765 reflections
*b* = 9.9900 (3) Å	θ = 2.7–28.3°
*c* = 11.2070 (3) Å	µ = 3.44 mm^−^^1^
α = 88.808 (1)°	*T* = 100 K
β = 83.098 (1)°	Platelet, brown
γ = 87.914 (1)°	0.54 × 0.32 × 0.12 mm
*V* = 627.25 (3) Å^3^	

### Data collection

**Table:**

Bruker APEXII CCD diffractometer	3084 independent reflections
Radiation source: fine-focus sealed tube	3004 reflections with *I* > 2σ(*I*)
graphite	*R*_int_ = 0.015
φ and ω scans	θ_max_ = 28.3°, θ_min_ = 1.8°
Absorption correction: multi-scan (*SADABS*; Bruker, 2008)	*h* = −7→7
*T*_min_ = 0.825, *T*_max_ = 1.000	*k* = −13→13
11074 measured reflections	*l* = −14→14

### Refinement

**Table:**

Refinement on *F*^2^	Primary atom site location: structure-invariant direct methods
Least-squares matrix: full	Secondary atom site location: difference Fourier map
*R*[*F*^2^ > 2σ(*F*^2^)] = 0.016	Hydrogen site location: inferred from neighbouring sites
*wR*(*F*^2^) = 0.042	H atoms treated by a mixture of independent and constrained refinement
*S* = 1.07	*w* = 1/[σ^2^(*F*_o_^2^) + (0.0186*P*)^2^ + 0.3513*P*] where *P* = (*F*_o_^2^ + 2*F*_c_^2^)/3
3084 reflections	(Δ/σ)_max_ = 0.001
179 parameters	Δρ_max_ = 0.56 e Å^−^^3^
0 restraints	Δρ_min_ = −0.28 e Å^−^^3^

### Fractional atomic coordinates and isotropic or equivalent isotropic displacement parameters (Å^2^)

**Table:**

	*x*	*y*	*z*	*U*_iso_*/*U*_eq_	
S1	0.75599 (5)	0.38684 (3)	0.03656 (3)	0.01156 (6)	
O1	0.88103 (16)	0.57056 (9)	0.18446 (8)	0.01639 (17)	
N1	0.35814 (18)	0.30153 (10)	0.13371 (9)	0.01243 (19)	
H71	0.234 (4)	0.2828 (19)	0.1871 (17)	0.030 (5)*	
N2	0.66139 (19)	0.65833 (10)	0.34986 (9)	0.01387 (19)	
H72	0.763 (3)	0.7156 (18)	0.3540 (16)	0.022 (4)*	
C1	0.5225 (2)	0.38913 (11)	0.15043 (10)	0.0116 (2)	
C11	0.6208 (2)	0.26265 (11)	−0.03624 (11)	0.0126 (2)	
C12	0.4045 (2)	0.22857 (12)	0.02886 (10)	0.0123 (2)	
C13	0.2600 (2)	0.13350 (12)	−0.01177 (11)	0.0149 (2)	
H13	0.1122	0.1115	0.0327	0.018*	
C14	0.3400 (2)	0.07239 (12)	−0.11923 (11)	0.0155 (2)	
H14	0.2447	0.0077	−0.1497	0.019*	
C15	0.5597 (2)	0.10425 (12)	−0.18438 (11)	0.0158 (2)	
H15	0.6115	0.0593	−0.2573	0.019*	
C16	0.7025 (2)	0.19979 (12)	−0.14454 (11)	0.0145 (2)	
H16	0.8503	0.2218	−0.1891	0.017*	
C21	0.6958 (2)	0.56953 (12)	0.25575 (11)	0.0130 (2)	
C22	0.5014 (2)	0.48055 (11)	0.24945 (10)	0.0120 (2)	
C23	0.2978 (2)	0.48983 (12)	0.33208 (11)	0.0138 (2)	
H23	0.1708	0.4313	0.3266	0.017*	
C24	0.2781 (2)	0.58459 (12)	0.42333 (11)	0.0152 (2)	
H24	0.1391	0.5909	0.4801	0.018*	
C25	0.4628 (2)	0.66773 (12)	0.42904 (11)	0.0151 (2)	
H25	0.4509	0.7333	0.4899	0.018*	
Br1	−0.08317 (2)	0.185566 (11)	0.318117 (10)	0.01427 (4)	
O90	0.0626 (2)	0.13628 (10)	0.59698 (10)	0.0246 (2)	
H901	0.039 (4)	0.139 (2)	0.527 (2)	0.044 (6)*	
H902	0.076 (4)	0.054 (2)	0.617 (2)	0.045 (6)*	

### Atomic displacement parameters (Å^2^)

**Table:**

	*U*^11^	*U*^22^	*U*^33^	*U*^12^	*U*^13^	*U*^23^
S1	0.01040 (12)	0.01293 (13)	0.01129 (13)	−0.00208 (10)	−0.00043 (10)	−0.00055 (10)
O1	0.0139 (4)	0.0194 (4)	0.0157 (4)	−0.0049 (3)	0.0005 (3)	−0.0020 (3)
N1	0.0114 (4)	0.0137 (5)	0.0121 (5)	−0.0021 (4)	−0.0003 (4)	−0.0001 (4)
N2	0.0150 (5)	0.0126 (5)	0.0144 (5)	−0.0033 (4)	−0.0025 (4)	−0.0003 (4)
C1	0.0107 (5)	0.0121 (5)	0.0120 (5)	−0.0002 (4)	−0.0020 (4)	0.0018 (4)
C11	0.0139 (5)	0.0114 (5)	0.0130 (5)	−0.0011 (4)	−0.0039 (4)	0.0008 (4)
C12	0.0135 (5)	0.0121 (5)	0.0114 (5)	0.0006 (4)	−0.0023 (4)	0.0004 (4)
C13	0.0137 (5)	0.0147 (5)	0.0163 (6)	−0.0017 (4)	−0.0023 (4)	0.0008 (4)
C14	0.0177 (6)	0.0135 (5)	0.0165 (6)	−0.0033 (4)	−0.0058 (5)	−0.0005 (4)
C15	0.0202 (6)	0.0153 (5)	0.0121 (5)	0.0004 (5)	−0.0028 (4)	−0.0014 (4)
C16	0.0144 (5)	0.0155 (5)	0.0133 (5)	−0.0001 (4)	−0.0006 (4)	0.0017 (4)
C21	0.0147 (5)	0.0128 (5)	0.0117 (5)	−0.0004 (4)	−0.0029 (4)	0.0014 (4)
C22	0.0130 (5)	0.0115 (5)	0.0118 (5)	0.0001 (4)	−0.0027 (4)	0.0003 (4)
C23	0.0132 (5)	0.0129 (5)	0.0155 (5)	−0.0008 (4)	−0.0020 (4)	0.0007 (4)
C24	0.0144 (5)	0.0153 (5)	0.0149 (5)	0.0007 (4)	0.0014 (4)	−0.0007 (4)
C25	0.0191 (6)	0.0126 (5)	0.0135 (5)	0.0011 (4)	−0.0019 (4)	−0.0012 (4)
Br1	0.01712 (7)	0.01291 (6)	0.01233 (6)	−0.00307 (4)	0.00086 (4)	−0.00021 (4)
O90	0.0405 (6)	0.0142 (5)	0.0219 (5)	−0.0057 (4)	−0.0138 (4)	−0.0005 (4)

### Geometric parameters (Å, °)

**Table:**

S1—C1	1.7215 (12)	C14—C15	1.4059 (18)
S1—C11	1.7461 (12)	C14—H14	0.9500
O1—C21	1.2379 (15)	C15—C16	1.3859 (17)
N1—C1	1.3304 (15)	C15—H15	0.9500
N1—C12	1.3887 (15)	C16—H16	0.9500
N1—H71	0.89 (2)	C21—C22	1.4469 (17)
N2—C25	1.3457 (16)	C22—C23	1.3885 (16)
N2—C21	1.3835 (15)	C23—C24	1.3998 (17)
N2—H72	0.832 (19)	C23—H23	0.9500
C1—C22	1.4429 (16)	C24—C25	1.3653 (18)
C11—C12	1.3949 (16)	C24—H24	0.9500
C11—C16	1.3987 (17)	C25—H25	0.9500
C12—C13	1.3924 (17)	O90—H901	0.81 (2)
C13—C14	1.3809 (17)	O90—H902	0.85 (2)
C13—H13	0.9500		
			
C1—S1—C11	90.47 (6)	C16—C15—C14	121.41 (11)
C1—N1—C12	115.00 (10)	C16—C15—H15	119.3
C1—N1—H71	124.5 (13)	C14—C15—H15	119.3
C12—N1—H71	120.2 (12)	C15—C16—C11	117.47 (11)
C25—N2—C21	124.55 (11)	C15—C16—H16	121.3
C25—N2—H72	116.9 (12)	C11—C16—H16	121.3
C21—N2—H72	118.2 (12)	O1—C21—N2	120.39 (11)
N1—C1—C22	123.82 (11)	O1—C21—C22	124.89 (11)
N1—C1—S1	112.33 (9)	N2—C21—C22	114.72 (11)
C22—C1—S1	123.78 (9)	C23—C22—C1	122.07 (11)
C12—C11—C16	120.69 (11)	C23—C22—C21	120.46 (11)
C12—C11—S1	110.79 (9)	C1—C22—C21	117.35 (10)
C16—C11—S1	128.53 (10)	C22—C23—C24	120.61 (11)
N1—C12—C13	126.76 (11)	C22—C23—H23	119.7
N1—C12—C11	111.41 (10)	C24—C23—H23	119.7
C13—C12—C11	121.83 (11)	C25—C24—C23	118.57 (11)
C14—C13—C12	117.41 (11)	C25—C24—H24	120.7
C14—C13—H13	121.3	C23—C24—H24	120.7
C12—C13—H13	121.3	N2—C25—C24	121.06 (11)
C13—C14—C15	121.18 (12)	N2—C25—H25	119.5
C13—C14—H14	119.4	C24—C25—H25	119.5
C15—C14—H14	119.4	H901—O90—H902	107 (2)
			
C12—N1—C1—C22	175.97 (10)	C12—C11—C16—C15	0.60 (17)
C12—N1—C1—S1	−0.99 (13)	S1—C11—C16—C15	−179.35 (9)
C11—S1—C1—N1	0.50 (9)	C25—N2—C21—O1	177.54 (11)
C11—S1—C1—C22	−176.46 (10)	C25—N2—C21—C22	−2.08 (17)
C1—S1—C11—C12	0.09 (9)	N1—C1—C22—C23	−4.11 (18)
C1—S1—C11—C16	−179.95 (11)	S1—C1—C22—C23	172.51 (9)
C1—N1—C12—C13	−178.24 (11)	N1—C1—C22—C21	179.71 (10)
C1—N1—C12—C11	1.06 (14)	S1—C1—C22—C21	−3.67 (15)
C16—C11—C12—N1	179.40 (10)	O1—C21—C22—C23	−178.23 (11)
S1—C11—C12—N1	−0.64 (12)	N2—C21—C22—C23	1.36 (16)
C16—C11—C12—C13	−1.25 (18)	O1—C21—C22—C1	−1.99 (18)
S1—C11—C12—C13	178.71 (9)	N2—C21—C22—C1	177.61 (10)
N1—C12—C13—C14	179.89 (11)	C1—C22—C23—C24	−176.56 (11)
C11—C12—C13—C14	0.66 (18)	C21—C22—C23—C24	−0.50 (18)
C12—C13—C14—C15	0.55 (18)	C22—C23—C24—C25	0.17 (18)
C13—C14—C15—C16	−1.19 (19)	C21—N2—C25—C24	1.87 (19)
C14—C15—C16—C11	0.59 (18)	C23—C24—C25—N2	−0.80 (18)

### Hydrogen-bond geometry (Å, °)

**Table:**

*D*—H···*A*	*D*—H	H···*A*	*D*···*A*	*D*—H···*A*
N1—H71···Br1	0.89 (2)	2.40 (2)	3.2708 (10)	168.2 (17)
N2—H72···O90^i^	0.832 (19)	1.930 (19)	2.7390 (15)	163.6 (17)
O90—H901···Br1	0.81 (2)	2.55 (2)	3.3485 (11)	170 (2)
O90—H902···Br1^ii^	0.85 (2)	2.49 (2)	3.3360 (10)	176 (2)

Symmetry codes: (i) −*x*+1, −*y*+1, −*z*+1; (ii) −*x*, −*y*, −*z*+1.
